# How to Validate a Bayesian Evolutionary Model

**DOI:** 10.1093/sysbio/syae064

**Published:** 2024-11-07

**Authors:** Fábio K Mendes, Remco Bouckaert, Luiz M Carvalho, Alexei J Drummond

**Affiliations:** Department of Biological Sciences, Louisiana State University, Baton Rouge, LA 70803, USA; School of Computer Science, The University of Auckland, Auckland 1010, New Zealand; Escola de Matemática Aplicada, Fundação Getulio Vargas, Rio de Janeiro, RJ 22250-900, Brazil; School of Biological Sciences, The University of Auckland, Auckland 1010, New Zealand

**Keywords:** Probabilistic model, Bayesian model, model validation, coverage

## Abstract

Biology has become a highly mathematical discipline in which probabilistic models play a central role. As a result, research in the biological sciences is now dependent on computational tools capable of carrying out complex analyses. These tools must be validated before they can be used, but what is understood as validation varies widely among methodological contributions. This may be a consequence of the still embryonic stage of the literature on statistical software validation for computational biology. Our manuscript aims to advance this literature. Here, we describe, illustrate, and introduce new good practices for assessing the correctness of a model implementation with an emphasis on Bayesian methods. We also introduce a suite of functionalities for automating validation protocols. It is our hope that the guidelines presented here help sharpen the focus of discussions on (as well as elevate) expected standards of statistical software for biology.

The last two decades have seen the biological sciences undergo a major revolution. Critical technological innovations, such as the advent of massive parallel sequencing and the accompanying improvements in computational power and storage, have flooded biology with unprecedented amounts of data ripe for analysis. Not only has intraspecific data from multiple individuals allowed progress in fields like medicine and epidemiology (e.g., Human Microbiome Project Consortium 2012; Neafsey et al. 2015; The 1000 Genomes Project Consortium 2015), population genetics (e.g., Lynch 2007; Lack et al. 2016; de Manuel et al. 2016), and disease ecology (e.g., Rosenblum et al. 2013; Bates et al. 2018), but now a large number of species across the tree of life have had their genomes sequenced, furthering our understanding of species relationships and diversification (e.g., Pease et al. 2016; Kawahara et al. 2019; Upham et al. 2019). Almost on par with data accumulation is the rate at which new computational tools are being proposed, as evidenced by journals entirely dedicated to method advances, methodological sections in biological journals, and computational biology degrees being offered by institutions around the world.

One extreme case is the discipline of evolutionary biology, on which we focus our attention. While it could be said that many decade-old questions and hypotheses in evolutionary biology have aged well and stood up the test of time (e.g., the Red Queen hypothesis, Van Valen 1973; Lively 1987; Morran et al. 2011; Gibson and Fuentes-González 2015; the Bateson—Dobzhansky–Muller model, Dobzhansky 1936; Muller 1940; Hopkins and Rausher 2012; Roda et al. 2017), data analysis practices have changed drastically in recent years, to the point they would likely seem exotic and obscure to an evolutionary biologist active 40 years ago. In particular, evolutionary biology has become highly statistical, with the development and utilization of probabilistic models now being commonplace.

Models are employed in the sciences for many reasons, and fall within a biological abstraction continuum (Servedio et al. 2014), going from fully verbal, highly abstract models (e.g., Van Valen 1973), through proof-of-concept models that formalize verbal models (e.g., Maynard Smith 1978; Reinhold et al. 1999), to models that interact directly with data through explicit mathematical functions (Yule 1924; Felsenstein 1973; Hasegawa et al. 1985; Hudson 1990). Within the latter category, probabilistic models have seen a sharp surge in popularity within evolutionary biology, in conjunction with computational tools implementing them.

Despite the increasing pervasiveness of probabilistic models in the biological sciences, tools implementing such models show large variation not only with respect to code quality (from a software engineering perspective) but also to the provided evidence for correctness (Darriba et al. 2018). This is unsurprising given the challenges in funding software research (Siepel 2019), and the multidisciplinary nature of method development. Much of the relevant information regarding good coding and statistical practices is out of reach of the average computational biologist, as it is spread across a variety of specialized sources, often obfuscated by its technical and theoretical presentation. The bioinformatics community is thus in dire need of synthetic and accessible resources that provide guidance for code improvement and validation.

Here, we summarize best practices in probabilistic model validation for method developers, with an emphasis on Bayesian methods. We execute two different validation protocols on variations of a simple phylogenetic model, discuss the results, and expand on how to interpret other potential outcomes. We further introduce a suite of methods for automating these protocols within the BEAST 2 platform (Bouckaert et al. 2019). Finally, we propose method development guidelines for new model contributions, for researchers, and reviewers who expect new software to meet not only a desirable standard but also a reasonable one.

## Probabilistic Models

Probabilistic models mathematically formalize natural phenomena having an element of randomness. This is done through probability distributions describing both the observed empirical data—seen as the result of one or more random instantiations of the modeled process—as well the model parameters, which abstract relevant, but usually unknown aspects of the phenomenon at hand. In the domain of evolutionary biology specifically, the historical, stochastic, and highly dimensional nature of evolutionary processes makes the utility of probabilistic models self-evident.

The central component of a probabilistic model, Pr(D=d|Θ=θ), allows us to describe the probability distribution over the data D (which takes value d) given the model parameters Θ (which take values θ). This probability mass function (pmf; or its continuous counterpart, the probability density function, pdf, $f_{D}(d|\Theta =\theta )$) is sometimes referred to as the likelihood function. Just for this section, we will abuse and simplify the notation for the image of $f_{D}$, and drop variable subscripts, for example, we will write $f_{D}(d|\Theta =\theta )$ as f(d|θ). As illustrated in the next sections, probabilistic models can be hierarchical, in which case there may be several likelihood functions. In a frequentist statistical framework, f(d|θ) is the sole component of an inferential procedure and is maximized across parameter space during parameter estimation and model comparison.

In the present study, we focus on Bayesian inference, where a probabilistic model ℳ defines a posterior probability distribution for its parameters, f(θ|d)=(f(d|θ)f(θ))∕f(d). Here, our prior inferences or beliefs about the natural world—represented by the prior distribution f(θ)—are confronted with and updated by the data through the likelihood function. Crucially, a Bayesian model includes a prior, f(θ): when models are compared, for example, f(θ) needs to be taken into account when computing the model evidence f(d).

Models routinely used in evolutionary biology are often characterized by continuous parameters, and are normally complex enough to preclude analytical solutions for the posterior density f(θ|d), mainly due to the intractability of the integral appearing in the denominator—that is, the marginal likelihood. In those cases, one can make use of the fact that f(d) is a constant with respect to the parameters that can be ignored (i.e., f(θ|d)∝f(θ|d)f(θ)), and use techniques like Markov chain Monte Carlo (MCMC) to sample (and hopefully converge on) the posterior distribution. This is because MCMC is usually implemented in the form of the Metropolis–Hastings (Metropolis et al. 1953; Hastings 1970) algorithm, which only requires the posterior to be evaluated up to a constant.

In practice, the Metropolis–Hastings algorithm samples the posterior distribution (also referred to as the “target” distribution) by means of a transition mechanism (i.e., a set of proposal functions). If the proposal distribution generated by this mechanism produces a Markov chain that is (i) irreducible (any “state,” or combination of parameter values, can be eventually reached from any other state), (ii) positive recurrent (there is an expected finite time for a state to be returned to), and (iii) aperiodic (every state has a period of 1, a requirement for most initial distributions to converge on the posterior distribution; see Levin and Peres (2017) for more details), and the chain is long enough, then the sampled posterior distribution will closely approximate the target distribution f(θ|d) (Smith and Roberts 1993; Tierney 1994; Gelman et al. 2013; we point interested readers to those references for more formal definitions).

We will spend time considering MCMC in particular, as it is the commonly chosen technique for obtaining samples from f(θ|d) under an implementation of model ℳ. A thorough validation effort thus entails verifying the correctness of (i) the model (i.e., f(d|θ)f(θ)), and (ii) the components involved in the MCMC transition mechanism. We note that the latter are not part of the model, however, and it is possible to sample f(θ|d) with other techniques such as importance sampling, Laplace approximations (Rue et al. 2009), or even by converting the sampling problem into an optimization one (e.g., Bouckaert 2024; Zhang and Matsen IV 2024).

Finally, we stress that we are interested in practices for verifying model correctness—and by “correctness,” we mean the correctness of a model implementation. There are other tests and diagnostics employed to ensure that a particular MCMC analysis is converging as expected. Ascertaining whether one or more independent Markov chains have converged to a given posterior distribution is not a correctness test, as that distribution might be very different from the target distribution. We refer the reader interested in these and related topics to Warren et al. (2017), Fabreti and Höhna (2022), Magee et al. (2023), and references therein.

## Validating a Bayesian Model

In this section, we discuss procedures for validating an implementation of a Bayesian model ℳ. Whenever necessary, we will differentiate between a model implemented as a simulator (S[ℳ]) and as a tool for inference (I[ℳ]). Both S[ℳ] and I[ℳ] must be inspected in order to validate a model ℳ.

### Validating the Simulator, S[ℳ]

When a new probabilistic model ℳ is introduced for the first time, a simulator for ℳ (S[ℳ]) must be devised and itself validated. The inferential engine (I[ℳ])—what users employ in empirical analyses—cannot be validated without a valid simulator. A simulator conventionally requires a parameter value as input (i.e., a value for Θ, θ, where θ might represent the values of more than one parameter), or a prior distribution on those values, $f_{\theta }(\cdot )$. Note that we use “⋅” when referring specifically to the generative function, rather than the value it takes given input. The simulator then outputs a sample of random variable(s), which for hierarchical models will include not only an instantiation d of data D but also of the parameters represented by Θ.

**Figure 1.**
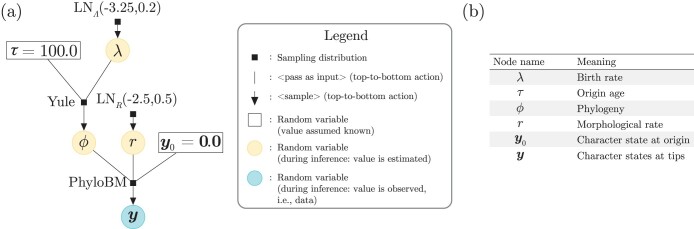
A simple probabilistic graphical (Bayesian) model is validated in this work. a) When read from top to bottom, the graphical model describes a generative process (see the legend for the meaning of vertical lines and downward-pointing arrows). If read from bottom to top, the graphical model describes the process of inference (assuming arrows having opposite orientation denoting the flow of information); in this case, the blue and yellow circles represent the data and the parameters being estimated, respectively. A random variable within a rectangular box signifies a parameter whose value is assumed known by the user; these are normally nuisance hyperparameters, or parameters that are not of immediate interest perhaps because they have been estimated elsewhere. b) Each random variable node in the model, and how they should be interpreted. Table 1 presents more detail on each of the sampling distributions. Briefly, “LN” stands for log-normal, “Yule” for a Yule process also known as a pure-birth model, and “PhyloBM” stands for a phylogenetic Brownian motion model.

In the case of hierarchical models, it is sometimes useful to consider S[ℳ] as a collection of component simulators, each characterized by a different sampling distribution. For example, the model ℳ we will work with below (Fig. 1; Table 1) consists of a hierarchical model; parametric distributions are used as hyperpriors (item 1, below), a Yule process is used as the phylogenetic tree prior (item 2), and phylogenetic Brownian motion is used as the data model (item 3):

$\text{S}[f_{\Theta }(\cdot )]$ (where $\Theta =\{T,\Lambda ,R,Y_{0}\}$), which jointly simulates $\theta =\{\tau ,\lambda ,r,y_{0}\}$,

$\text{S}[f_{\Phi |T,\Lambda }(\cdot |T=\tau ,\Lambda =\lambda )]$ , which simulates a Yule-tree ϕ given an origin age value τ and a λ (the birth rate) simulated in (1),

$\text{S}[f_{Y|\Phi ,R,Y_{0}}(\cdot |\Phi =\phi ,R=r,Y_{0}=y_{0})]$ , which simulates an array with k continuous-trait values (one value per species), y, given a phylogeny ϕ with s species, an evolutionary rate r, and ancestral character values $y_{0}$ (simulated in (1) and (2), respectively).

**Table 1:** Sampling distributions used in the probabilistic model validated in this work (Fig. 1).

Label (Fig. 1)	Full name or alias	During simulation	During inference
${\text{LN}}_{\Lambda }(-3.25,0.2)$	Log-normal	$f_{\Lambda |{ℳ}_{\Lambda },\Sigma_{\Lambda }}(\cdot |{ℳ}_{\Lambda }=-3.25,\Sigma_{\Lambda }=0.2)$	$f_{\Lambda |{ℳ}_{\Lambda },\Sigma_{\Lambda }}(\lambda |{ℳ}_{\Lambda }=-3.25,\Sigma_{\Lambda }=0.2)$
${\text{LN}}_{R}(-3.25,0.2)$	Log-normal	$f_{R|{ℳ}_{R},\Sigma_{R}}(\cdot |{ℳ}_{R}=-2.5,\Sigma_{R}=0.5)$	$f_{R|{ℳ}_{R},\Sigma_{R}}(\lambda |{ℳ}_{R}=-2.5,\Sigma_{R}=0.5)$
Yule	Pure-birth	$f_{\Theta |T,\Lambda }(\cdot |T=\tau ,\Lambda =\lambda )$	$f_{\Theta |\Lambda }(\lambda |T=\tau ,\Lambda =\lambda )$
PhyloBM	Phylogenetic Brownian motion	$f_{Y|\Theta ,R,Y_{0}}(\cdot |\Theta =\theta ,R=r,Y_{0}=y_{0})$	$f_{Y|\Theta ,R,Y_{0}}(y|\Theta =\theta ,R=r,Y_{0}=y_{0})$

Notes: Columns “During simulation” and “During inference” specify how the sampling distributions should be read and interpreted, following the notation in the main text.

Being able to isolate the building blocks of a hierarchical model simulator helps divide and conquer the validation task, especially when some but not all of the sampling distributions are well-known parametric distributions, or when they result from well-characterized stochastic processes (see below).

One way to validate a probabilistic model simulator is by using it to produce (sample) a large number of data sets given a set of parameters. For each data set, one can then construct α×100%-confidence intervals (where α∈(0,1) gives the confidence level) for certain summary statistics (e.g., mean, variance, covariance). If the simulator is behaving as expected, one should be able to verify that the (ensemble’s or “true”) summary statistic is contained approximately α% of the time within their α×100%-confidence intervals. An example is the Yule model (also known as the pure-birth model; Yule 1924), a continuous-time Markov process that has been classically employed in phylogenetics to model the number of species in a clade (Yule 1924; Aldous 2001). Under a Yule process with a species birth rate of λ, the expected tree height, $\text{E}[t_{\text{root}}]$, for a tree with s tips is:


$$\begin{array}{c} \text{E}[t_{\text{root}}]=\overset{s}{\underset{i=2}{\sum }}\frac{1}{i\lambda }. \end{array}$$ (1)

**Figure 2.**
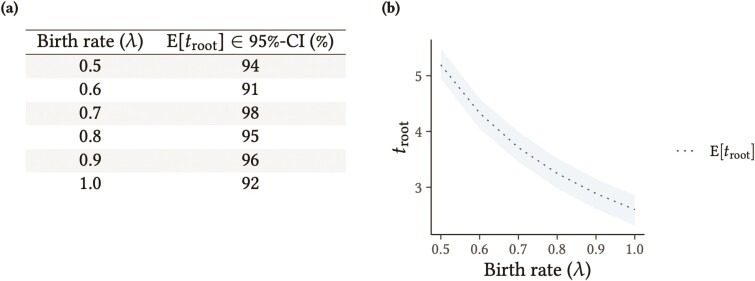
Validation results for a Yule model simulator. (a) How often the expected Yule-tree root age fell within its 95%-confidence intervals, for different birth rates (see main text for more details). (b) Graphical representation of table in (a).

One can then verify, for example, if $\text{E}[t_{\text{root}}]$ is 95% of the time within ±1.96 standard errors of the average Yule-tree root age (from each sampled data set). Confirming that this is the case indicates $\text{S}[f_{\Phi |\Lambda }(\cdot |T=\tau ,\Lambda =\lambda )]$ is correctly implemented (Fig. 2). Interested readers will find another example of this validation procedure in the supplement, where the density function characterizing the model (a phylogenetic Brownian motion model, “PhyloBM”; Felsenstein 1973) is that of a parametric distribution, namely the multivariate normal. Protocols for validating I[ℳ] (see below) will also normally validate S[ℳ] at the same time.

We note that we have so far used S[ℳ] to represent a *direct* simulator under model ℳ, meaning each and every sample generated by S[ℳ] is independent. (Researchers wishing to carry out direct simulation will find many examples of capable software in Supplementary Table S1.) This is in contrast with other simulation strategies, such as conducting MCMC under model ℳ with no data, that is, “sampling from the prior,” given specific parameter values (θ). This latter approach may be the only option if S[ℳ] has not been yet implemented, and it is predicated upon the existence of correct implementations of both an inferential engine $\text{I}{\left[ℳ\right]}^{\prime }$ and of proposal functions. We distinguish $\text{I}{\left[ℳ\right]}^{\prime }$ from I[ℳ] because simulations are being carried out precisely to validate I[ℳ]. Unless MCMC simulations are done with $\text{I}{\left[ℳ\right]}^{\prime }$—an independent and validated implementation of I[ℳ]—they can introduce circularity to the validation task.

### Validating the Inferential Engine, I[ℳ]

The more complex the natural phenomenon under study, the more difficult it will be to strike a good balance between model practicality and realism (Levins 1966). The popular aphorism rings true: “all models are wrong but some are useful” (Box 1979). Very simple models are easier to implement in efficient inference tools, but will commonly make assumptions that are likely to be broken by the data. Conversely, complex models will fit the data better but may become unwieldy with increasing levels of realism.

A large number of parameters can cause overfitting and weak identifiability, and inference under highly complex models might be prohibitively slow (Shapiro et al. 2000). Deciding on the utility of a model for real-world problems is a daunting task (Brown and Thomson 2018; Shepherd and Klaere 2018), and is a challenge we do not address in the present contribution. Such model appraisals (what we call “model characterization” below) are normally carried out after a model is published, often in multiple contribution bouts, and are critical for a model’s longevity. Analyses of model fit against data are normally accompanied by discussions on assumption validity, and more rarely by benchmarking and scrutinization of model behavior and implementation (e.g., Maddison et al. 2007; Stadler 2010; Rabosky et al. 2013; Rabosky and Goldberg 2015; Moore et al. 2016).

When a new model ℳ is initially proposed, however, authors must ensure that their methods can at the very least robustly recover generating parameters. In this section, we discuss a few techniques that can be employed to assess the correctness of a parameter-estimation routine. These techniques assume that one can accurately simulate from a probabilistic data-generating process (see section Validating the Simulator, S[ℳ]).

#### Coverage validation

**Figure 3.**
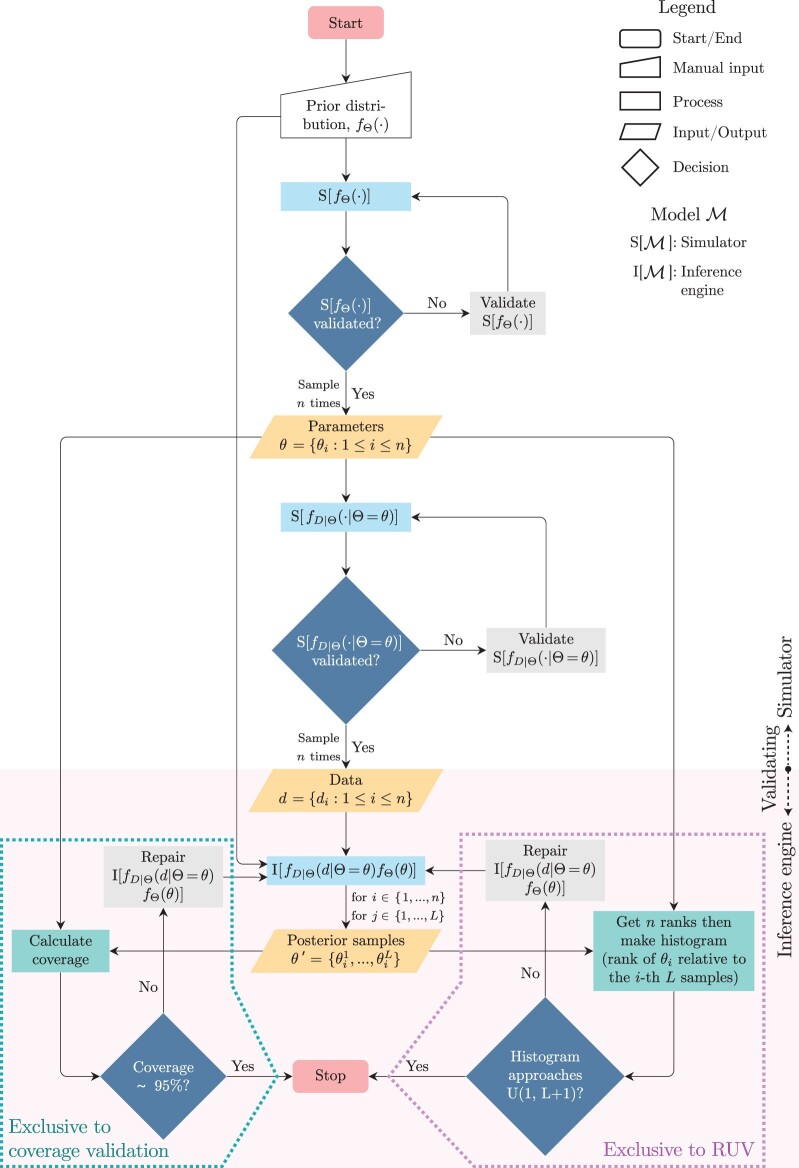
Flowchart of the validation of a Bayesian model. Standard flowchart symbols are explained in the legend. The flowchart area with a clear background is where (true) parameters and data are generated, and where the model simulator(s) is validated. The flowchart area shaded in pink marks the steps involved in validating the inference engine once the data has been generated. θ denotes a vector with n elements, where each element is an i.i.d. parameter(s) sample from its (their) corresponding prior(s), $f_{\Theta }(\cdot )$. Analogously, d denotes a vector with n elements, where each element is an i.i.d. data sample from the corresponding likelihood(s) $f_{D|\Theta }(\cdot |\Theta =\theta )$. $\theta^{\prime }$ holds n×L elements, with each being one of the L posterior samples for each of the n parameter samples in θ. All L posterior samples obtained from the ith data set $d_{i}$ comprise together what one would call the posterior distribution over $\theta_{i}$. Posterior samples are commonly obtained through MCMC. U(l,u) denotes a uniform distribution with and including lower and upper bounds l and u, respectively. The aquamarine dotted box encloses the stages of the pipeline that are exclusive to the coverage validation procedure. The pink dotted box encloses the stages of the pipeline that are exclusive to the rank-uniformity validation (RUV) procedure.

Our discussion on how to ensure a Bayesian model is well calibrated and thus correct will mostly follow the ideas in Cook et al. (2006) and Talts et al. (2018). The basic idea is presented in the flowchart in Figure 3 (acquamarine dotted box and what is above it), and consists of 3 stages, namely simulation, inference, and coverage calculation. Before we delve into more details, the coverage of a parameter is simply how often the (true or simulated) parameter value falls within an estimated Bayesian highest posterior density (HPD) interval. What constitutes “acceptable” coverage, however, depends on the researcher’s desired credibility level, α. In what follows, we formally introduce this and other terms and describe a protocol for assessing whether coverage is appropriate.

Let us assume we have a validated simulator for model ℳ, and now it is time to validate ℳ’s inferential engine. We will start by sampling n parameter sets $\theta =\{\theta_{i}:1\leq i\leq n\}$ from its prior, $f_{\Theta }(\cdot )$, that is:


$$\begin{array}{llll} \theta_{i} & \sim f_{\Theta }(\cdot ). & & \end{array}$$

For each parameter set $\theta_{i}$, we then sample a data set $d_{i}$ from $f_{D|\Theta }(\cdot |\Theta =\theta_{i})$:


$$\begin{array}{llll} d_{i} & \sim f_{D|\Theta }(\cdot |\Theta =\theta_{i}), & & \end{array}$$

These two steps conclude the “simulation” stage of this validation protocol. With $d=\{d_{i}:1\leq i\leq n\}$ , we use the inferential machinery I[ℳ] under evaluation to compute $f_{\Theta |D}(\theta_{i}|D=d_{i})$ for each $d_{i}$. Recall that we assume the posterior distribution defined by $f_{\Theta |D}(\theta |D=d)$ over Θ will be approximated with MCMC, an algorithm that generates a large sample of size L of parameter values from that posterior distribution, $\theta^{\prime }=\{\theta_{i}^{j}:1\leq i\leq n,1\leq j\leq L\}$. At this point, we have concluded the inference stage of this validation pipeline.

The third stage and final stage consists of investigating coverage properties of uncertainty intervals. The critical expectation here is that if the inferential engine is correct, we will be able to obtain interval estimates with precise coverage properties. More concretely, let us first define the HPD interval. For a credibility level α∈(0,1), we define $I_{\alpha }(d):=(a(d,\alpha ),b(d,\alpha ))$ such that:


$$\frac{1}{f_{D}(d)}\int_{a(d,\alpha )}^{b(d,\alpha )}f_{D|\Theta }(d|\Theta =\theta )f_{\Theta }(\theta )d\theta =\alpha ,$$

where $f_{D}(d)$ is a constant that can be ignored. By defining $\text{Cred}(I_{\alpha }(d))=\alpha$,


$$\underset{b(d,\alpha )-a(d,\alpha )}{\text{inf}}\left\{I_{\alpha }(d):\text{Cred}(I_{\alpha }(d))=\alpha \right\}$$

yields the shortest interval with the required credibility. Note that we approximate a particular $I_{\alpha }(d_{i})$ from the ith L samples obtained with MCMC, in $\theta^{\prime }$.

Now taking a set of parameter values $\theta_{i}$ sampled from $f_{\Theta }(\cdot )$ it can be shown that $\text{ Pr}\left(\theta_{i}\in I_{\alpha }(d)\right)=\alpha$, that is, that 100×α% HPDs have nominal coverage under the true generative model (a proof for any α is provided in the supplementary material). More formally, the coverage of n intervals obtained as above will be distributed as binomial random variable with n trials and success probability α. When n=100 and α=0.95, the 95%-central interquantile interval for the number of simulations containing the correct data-generating parameter is between 90 and 99 (Table 2 shows the interquantile intervals for other α values.). If we ascertain that I[ℳ] of a Bayesian model produces coverage lying within the expected bounds, we say the model has passed the coverage validation, and is well calibrated and correct.

**Table 2:** The 95% central interquantile intervals for the number of HPD intervals covering the true parameter value (obtained during coverage validation), under different credibility levels and numbers of replicates.

Credibility level % (100×α)	n (replicates)	Lower quantile	Upper quantile
	100	40	60
50	200	86	114
	500	228	272
	100	66	83
75	200	138	162
	500	356	394
	100	84	95
90	200	171	188
	500	436	463
	100	90	99
95	200	184	196
	500	465	484
	100	97	100
99	200	195	200
	500	490	499

Notes: Assuming model correctness, the number of true simulated values that fall within their corresponding 100 × α%-HPDs (coverage) is binomially distributed with *n* trials and probability of success α.

At this point, we will take a moment to remark that the usefulness of model coverage analysis in Bayesian inference is only manifest when $\theta_{i}$ is sampled from $f_{\Theta }(\cdot )$. Method developers may be tempted, for example, to calculate coverage for specific parameter values—perhaps chosen across a grid over parameter space—using a different prior during inference. In such cases, we emphasize that obtaining a coverage lower than 95% (for 95% HPDs) does not necessarily mean that a model is incorrectly implemented; conversely, obtaining exactly 95% coverage does not imply model correctness. Coverage values only have bearing on model correctness if, and only if, random variables are sampled from the same prior distribution used in inference.

**Figure 4.**
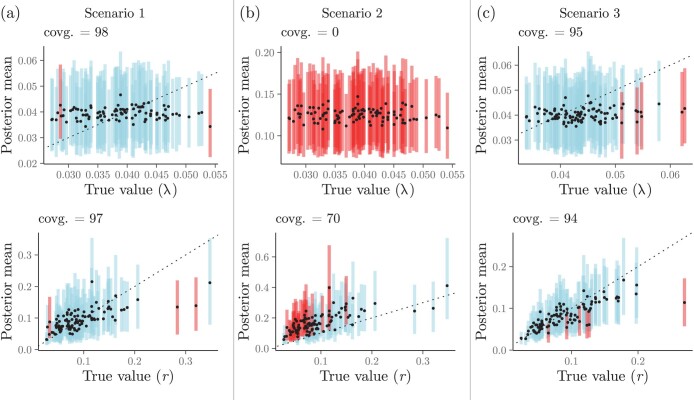
Coverage validation analyses of the Bayesian hierarchical model in Figure 1. Panels show the true (i.e., simulated) parameter values plotted against their mean posteriors (the dashed line shows x=y). Dots and lines (100 per panel) represent true values and their 95%-HPDs, respectively. Simulations for which 95%-HPDs contained the true value are highlighted in blue, otherwise are presented in red. a) In “Scenario 1,” model used in inference was the least misspecified (low levels of misspecification were introduced by rejection sampling when one in 10 trees was rejected). b) In “Scenario 2,” the model used in inference was misspecified beyond the effect of rejection sampling (which was the only source of misspecification in “Scenario 1” and “Scenario 3”); here, we used the same data sets simulated in “Scenario 1.” c) In “Scenario 3,” the model was misspecified as a result of rejection sampling as in “Scenario 1,” with the difference that a greater proportion of trees were rejected (approximately 90% of trees were rejected, with only those having between 100 and 200 tips being kept).

We provide examples of coverage validation attempts in Figure 4, which shows coverage graphical summaries for data simulated under the model represented in Figure 1. This model is deliberately simple for the sake of brevity and clarity in the discussion below. The parameters in this model are the phylogenetic tree Φ, the species birth rate Λ, and the continuous-trait evolutionary rate R (we assume the continuous-trait value at the root, $Y_{0}$, is known and set it to 0.0 for all simulated data sets). When the model is correctly specified, or very close to being correctly specified between simulation and inference (“Scenario 1”, Fig. 4a), coverage is close to 95% and adequate for both Λ and R, which indicates that I[ℳ]—as implemented in BEAST 2, the software we used—is well calibrated and correct.

In “Scenario 2” of Figure 4b, however, we misspecify the model during inference, setting the prior distribution on Λ to be a log-normal with a mean of -2.0 (rather than -3.25, as specified in the simulation procedure; Fig. 1). In contrast with scenario 1, coverage is 0.0 for Λ and 70% for R, both much lower and outside the expected coverage bounds (Table 2). These numbers indicate that one or more of the parts comprising model ℳ used in I[ℳ] differs from their counterparts in S[ℳ]. This result was expected because we purposefully made the models in simulation and inference differ; we know I[ℳ] is correct because of the results from scenario 1. Of course, in a real-world validation experiment, the model should be correctly specified, and such a result would suggest a problem with the inferential machinery (provided the simulator had been previously validated).

Finally, in “Scenario 3” of Figure 4c we specified the model just like in Scenario 1 but carried out substantial rejection sampling during simulation. Approximately 90% of all simulated trees were rejected based on their taxon count; trees were rejected if they had fewer than 100 or more than 200 taxa. As with Scenario 1, coverage fell within the expected ranges for a correct model implementation. This result may strike the reader as odd: if I[ℳ] expects trees with a wide range of tip numbers, and we feed it simulated trees within a narrow tip number interval, should this not lower coverage? For example, one may have expected the estimated λ to be consistently higher or lower than the true λ. Λ is nonetheless challenging to infer under the current model, as suggested by estimates falling around the corresponding prior mean value; unlike in Scenario 2, however, here the prior mean parameter was correctly specified. As a result, coverage validation was not capable of detecting any symptoms arising from the rejection of tree samples.

Scenario 3 brings home the point that an incorrect model implementation may pass coverage validation unless model misspecification is sufficiently severe (e.g., Scenario 2), or parameter estimate location is highly responsive to the evidence in the data—unlike Λ in the examined model. (In the supplement we expand on this point using a different and simpler model and show that, if extreme, rejection schemes will be detected by coverage validation as a model misspecification issue; Supplementary Table 3.) Put simply, obtaining appropriate coverage may not be enough to ascertain that a model is correct. Potential biases in parameter estimates may remain undetected unless more investigation is done (see “Rank-uniformity validation” section).

The three scenarios we explored above illustrate how coverage validation results can be interpreted in terms of model implementation correctness. One can additionally capitalize on this validation setup and gauge how accurate an inferential tool can be for different parameters. The easier it is to estimate a parameter, the higher should be the correlation between its posterior mean and its generating “true” value. In our Scenarios 1 and 3, the species birth rate Λ was hard to estimate given the sizes of the phylogenetic trees. Conversely, the continuous-trait evolutionary rate, R, was more easily identifiable, as revealed by the higher correlation between its true values and their posterior means. We conclude this section by noting that the absence of correlation between parameter estimates and their true values (sometimes referred to as “weak unidentifiability”) should not be taken as a sign that a model is incorrect—inappropriate coverage values should.

#### Rank-uniformity validation

**Figure 5.**
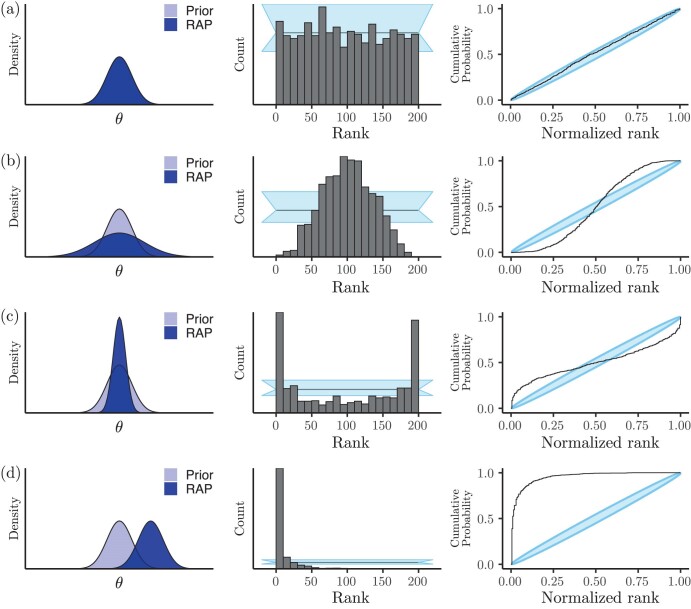
Patterns observable after inference in RUV. We explain how to interpret the histogram of ranks (middle column) and ECDF plots (right-hand side column) in the main text. a) Model implementation is correct. b) Parameter estimates are overdispersed relative to their true values. c) Parameter estimates are underdispersed relative to their true values. d) Parameter estimates are consistently overestimated relative to their true values. In the left-hand side column, the prior and replicate-averaged posterior (RAP; also known as the data-averaged posterior) distributions over some parameter θ are shown in light blue and dark blue, respectively. In the middle graphs, light-blue bands represent the 95%-confidence interval about the expected rank count, and horizontal black lines mark the rank count mean. Light-blue ellipses in the rightmost graphs represent confidence intervals about the ECDF.

Talts et al. (2018) showed that one can devise other tests that might be more powerful to detect problems than just looking at the coverage of Bayesian HPD intervals. In particular, given $\theta =\{\theta_{i}:1\leq i\leq n\}$ (produced according to the protocol in Fig. 3), those authors demonstrated (Theorem 1 therein) that if the inference machinery I[ℳ] works as intended, the distribution of the rank $r_{i}$ of $\theta_{i}$ ($\theta_{i}$ being the ith parameter draw out of n random draws from its prior distribution) relative to $\theta_{i}^{\prime }$ —that is, the rank of the ith parameter value relative to its corresponding L MCMC chain samples— will follow a uniform distribution on [1,L+1] (Fig. 3, pink dotted box; Fig. 5a). In other words, if one were to sort all true parameter values $\theta_{i}$ against $\theta_{i}^{\prime }$ —their corresponding L MCMC posterior samples—the first (smallest ranking) 10% out of n$\theta_{i}$ values should account for approximately 10% of the total rank mass; the next 10% of (higher ranking) $\theta_{i}$ values should account, again, for approximately 10% of the total rank mass, and so on.

Adherence to this distribution can be investigated by constructing histograms (Talts et al. 2018) as well as by looking at the empirical cumulative distribution function (ECDF) and their confidence bands (Säilynoja et al. 2022). When a model implementation fails RUV, it can do so in different ways. For instance, when the inference machinery leads to consistent overdispersed estimates, it produces a pattern of ranks concentrating around the middle rank (Fig. 5b). When underdispersion is present, on the other hand, ranks tend to bunch up towards the ends (Fig. 5c), creating a pattern of “horns,” which can also be caused by high autocorrelation in the MCMC draws. This is also why we recommend thinning MCMC draws in order to reduce autocorrelation. Figure 5d shows the rank patterns when the inference machinery produces biased estimates: ranks will bunch up against one of the ends, depending on whether estimates are biased downwards or upwards. In the particular case shown in Figure 5d, the parameter at hand is being overestimated.

**Figure 6.**
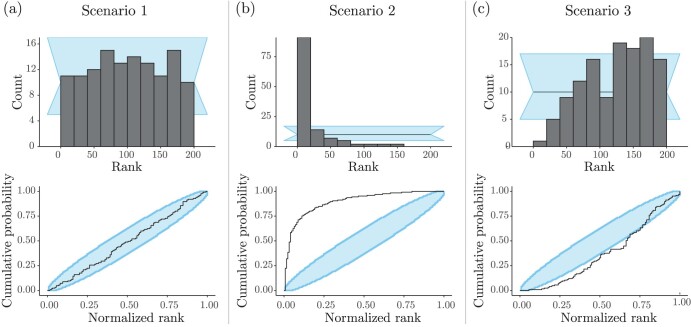
RUV of the Bayesian hierarchical model in Figure 1. Panels in the top row show the histograms of n=100 ranks, for parameter R in each scenario, obtained after 10% burnin and thinning of posterior samples down to 200 out of 10,000. Panels in the bottom row show the corresponding ECDF plots, for parameter R in each scenario. a) In “Scenario 1,” the inference model was the least misspecified (low levels of misspecification were introduced by rejection sampling) and we can see that the ranks are compatible with a uniform distribution (within the blue band). b) In “Scenario 2”, the inference machinery was misspecified beyond the effect of rejection sampling (which was the only source of misspecification in “Scenario 1” and “Scenario 3”); here, we used the same data sets simulated in “Scenario 1.” A clear pattern of overestimation shows up in the ranks, meaning the ranks for the data-generating values are usually smaller than expected under correctness. c) In “Scenario 3” we can see a pattern of underestimation, evidenced by ranks bunching up to the right. Rejection sampling was more extreme in this scenario (i.e., a more misspecified model in inference) than in that shown in a).

We conducted RUV on the three scenarios described in the previous section, which makes use of the model depicted in Figure 1. In the interest of brevity, we only show the histograms and ECDFs for R, and leave the remaining plots for Λ to the supplement. As expected, under Scenario 1, our model implementation passes the RUV—as indicated by histogram bars and ECDF values falling within their 95%-confidence intervals (Fig. 6a).

Under Scenario 2, again as expected, our method failed RUV (Fig. 6b). In particular, we observed a great overestimation of the Brownian motion rate (R). In a real-world analysis, these results would point to one or more faulty implementations (e.g., one or more model components, MCMC machinery, the simulator, etc). We remind the reader that in our experiment, Scenario 2 was purposefully set up so that the (prior) models used in simulation and inference differed; our implementations are actually correct, but were induced to fail the RUV procedure.

Finally, RUV results for Scenario 3 contrasted with what we observed for this scenario’s coverage validation (Fig. 4c). While the model specified in Scenario 3 passed its coverage validation (coverage was acceptable for both Λ and R), it did not pass the RUV procedure. The corresponding rank histogram and ECDF plots indicate that R is underestimated (Fig. 6c). This result suggests that RUV can be more sensitive than coverage validation, at least for certain types of model misspecification, such as those affecting parameter estimate location.

Unlike Scenario 2, in which we caused an explicit mismatch between the distributions used in simulation and inference, model misspecification under Scenario 3 was subtler: simulation and inference models were identical (as in Scenario 1), but tree samples from $f_{\Phi }(\cdot )$ (the Yule prior) were often rejected as θ was generated. The model used in Scenario 3 failed RUV because rejecting tree samples induced an implicit Yule model in simulation that differed from the Yule model used in inference. Indeed, using a much simpler model and an analogous rejection scheme (Supplementary Fig. 3), we were able to recapitulate the results in Figure 6.

### Tree Models

Tree models are stochastic processes that can capture the most fundamental tenet in evolutionary biology, namely common descent, at multiple time scales. Over the last few decades, pivotal theoretical work has not only characterized many properties of the more elementary tree models (for examples and an overview, see Nee 2006; Wakeley 2009; Stadler 2013; Harmon 2018) but also generalized them to be more realistic. Popular among empiricists, for example, are tree models that allow for lineage-affecting event rates that vary over time and across taxa, and that are state-dependent (“state” here meaning the attributes of a lineage’s genotypic, phenotypic, ecological, or biogeographic characters). Such models lend themselves to the study of evolutionary phenomena such as species diversification and infectious disease spread.

Although convenient evolutionary abstractions, tree models can nonetheless be challenging to formalize depending on their level of realism. The parameter space of a tree model is difficult to handle: it includes both a combinatorially complicated discrete component (the tree topology) and a continuous component (the branch lengths) (Semple et al. 2003). The theoretical properties, summarization, and exploration of tree space are all active topics of research in mathematical and computational biology (Gavryushkina et al. 2013; Gavryushkin and Drummond 2016; Brown et al. 2020).

Given the interest in tree models shown by empirical, computational, and theoretical biologists, in this section we will cover how tree models have been and can be validated, with an emphasis on tree space. We also propose two new ways in which tree models can undergo RUV and be assessed with respect to coverage, respectively. Our treatment is not meant to be an exhaustive review, but a short synthesis, and in keeping with the subject of the present work, we will not discuss protocols for the development and validation of Bayesian proposals in tree space. This topical subject is multifaceted (e.g., Douglas et al. 2021; Bouckaert 2022; Douglas et al. 2022) and deserves a dedicated contribution we leave for the future.

One way a tree model implementation can and has been validated is by comparing statistical summaries of its samples (drawn through direct simulation or MCMC without data) against theoretical “target” values (e.g., Fig. 2). This type of validation can be compelling and is often easy to carry out, but closed-form expressions tend to be only available for simpler models like the birth–death process, the Kingman’s coalescent, and a few of their special cases and generalizations. Such expressions remain useful, nonetheless, as long as more complex models can be constrained to forms for which the relevant theory exists. Typical theoretical targets include the first moments of distributions on tree characteristics such as internal node ages, internal, and terminal branch lengths, the sum of branch lengths, the number of tips, and the frequency of different tree topologies (Tajima 1983; Rosenberg 2002; Aldous and Popovic 2005; Gernhard 2006; Nee 2006; Gernhard 2008; Wakeley 2009; Mooers et al. 2012).

As tree models increase in complexity (e.g., Maddison et al. 2007; Fitzjohn 2010; Goldberg and Igić 2012; Sciré et al. 2022), so does the tree space they define and as a consequence theoretical model validation as described above becomes difficult. When nodes can be serially sampled or direct ancestors of other nodes, for example, even enumerating all the possible trees under a model is a non-trivial exercise (Gavryushkina et al. 2013). The number of tips in a tree can also complicate the theoretical characterization of tree models; except for when the number of tips is small (Drummond and Bouckaert 2015), algorithms have to be employed to generate expectations from theoretical principles (Kim et al. 2019).

When theoretical predictions useful for validation cannot be made, an often-employed method involves the comparison of independent model implementations, with one or both being simulators or inference engines. Tree samples from a direct simulator can be compared to samples drawn with MCMC without data (e.g., Zhang et al. 2024), or exact likelihood values can be compared between different implementations (Andréoletti et al. 2022). On extreme cases, however, even that strategy appears unattainable. For example, if it is unclear how to even directly simulate under a tree model—such as when node-age prior distributions are added to a birth–death process (models used in “node-dating,” Ho and Phillips 2009; but see Heled and Drummond 2012)—there seems to be no discernible path for validation in tree space.

**Figure 7.**
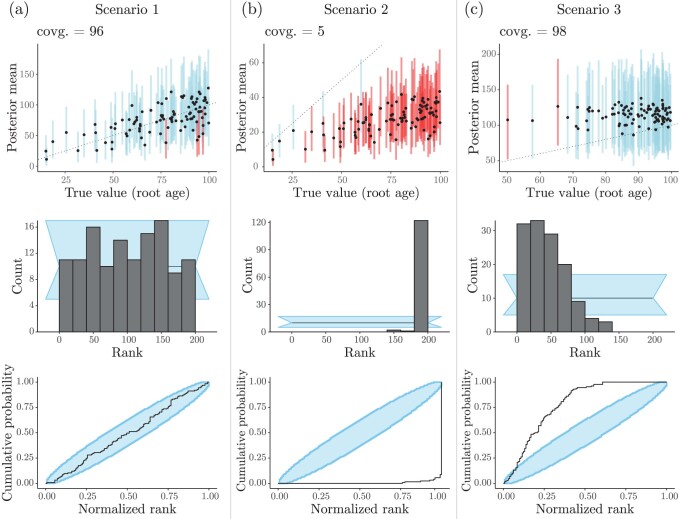
Coverage validation and RUV of the Bayesian hierarchical model in Figure 1, for each scenario described in the main text (also see Figs. 4 and 6), with respect to the height of ϕ (i.e., the phylogeny’s root age). Panels in the top row show the true (i.e., simulated) root age values plotted against their mean posteriors (the dashed line shows x=y). Dots and lines (100 per panel) represent true values and their 95%-HPDs, respectively. Simulations for which 95%-HPDs contained the true value are highlighted in blue, otherwise are presented in red. Panels in the middle row show the RUV histograms of n=100 ranks in each scenario, obtained after 10% burnin and thinning of posterior samples down to 200 out of 10,000. Panels in the bottom row show the corresponding RUV ECDF plots in each scenario.

The procedures of coverage validation and RUV can also be used to examine parameters in tree space. So far authors have mainly focused on the coverage of quantities such as species- and gene-tree root ages, sum of branch lengths, and number of direct ancestors (e.g., Gavryushkina et al. 2014; Ogilvie et al. 2022; Zhang et al. 2024). Figure 7, for example, shows the coverage of the root age for the three scenarios we explored in the previous sections. Similarly to what was observed for tree-unrelated parameters, the root age had the expected coverage in Scenarios 1 and 3, and RUV again aligned with the diagnosis of model misspecification for Scenario 3.

To the best of our knowledge, the RUV procedure has not been applied to validate phylogenetic models *vis-a-vis* tree space. This is likely because, due to the complexity of tree space, it is hard to canonically rank labeled trees such as the ones we focus on here. For the reasons above, while many studies have fine-combed the behavior of tree models in a variety of ways, experiments in coverage validation of tree topologies are particularly rare. As mentioned earlier, experiments of the kind do exist, but have been limited to specific dimensions of phylogenetic space (e.g., Gavryushkina et al. 2014; Heath et al. 2014; Ogilvie et al. 2022; Zhang et al. 2024; see also Höhna 2020 and references in Höhna and May 2022). Total ordering of unlabeled topologies (shapes) is nonetheless possible (Colijn and Plazzotta 2018; Maranca and Rosenberg 2024) and can be employed when running RUV on this space. Here, we propose further ways of mapping labeled trees to the real line and compute ranks. In what follows we introduce two novel approaches: (i) a solution for ranking trees as a part of a RUV analysis, and (ii) a way in which the topology of a tree can have its coverage assessed.

For the first method, we propose that each of the trees in a set of MCMC samples, as well as the corresponding “true” tree, be first compared to an external, “reference” tree sampled randomly from the prior (Algorithm 1 in the supplement describes the whole procedure). For example, a tree sample can be compared to the reference tree with respect to the length of their longest branch, to their topology, to their asymmetry, and so on; what matters here is that this comparison quantitatively measures the distance between the reference and the other tree. Then, once one knows how distant each true tree and its posterior samples are from the reference tree, they can be ranked relative to one another based on their associated distance measure. RUV proceeds normally from this point.

**Figure 8.**
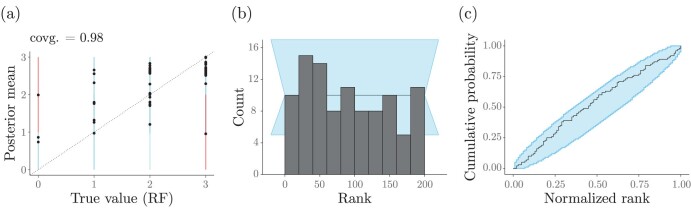
Coverage validation and RUV of a Kingman’s coalescent model with respect to the Robinson–Foulds distance (RF; see text in box) between the coalescent tree (Φ) and a reference (random) tree ($\phi_{0}$). The effective population size parameter is assumed known and fixed to 1.0 during inference. a) The true RF distances (i.e., between 100 simulated coalescent trees, $\phi =\{\phi_{i}:1\leq i\leq 100\}$, and the same reference tree, $\phi_{0}$) plotted against the corresponding mean posterior RF distances (calculated from posterior samples of each $\phi_{i}$ and $\phi_{0}$). The dashed line shows x=y. Dots and vertical lines represent true RF-distance values and their estimated 95%-HPDs, respectively. Simulations for which 95%-HPDs contained the true value are highlighted in blue, otherwise are presented in red. b) RUV histograms of 100 ranks obtained after 10% burnin and thinning of posterior samples down to 1000 out of 10,000. c) RUV ECDF plots.

We illustrate RUV in tree space using a specific functional, or phylogenetic metric, the Robinson–Foulds distance (RF; Robinson and Foulds 1981) between two trees, which counts the number of clades implied by one tree but not the other. For our purposes, computing the RF distance requires having a reference phylogeny $\phi_{0}$ to which we can compare our focal generating phylogeny ϕ and its posterior MCMC samples. The RUV protocol remains the same, with an additional step in which we generate $\phi_{0}$; results for five-taxon Kingman coalescent trees (under a known effective population size of 1.0) can be seen in Figure 8. Figure 8a shows the coverage of 95%-HPD intervals of the RF distance metric, while Figure 8b and c give its rank distribution and empirical cumulative distribution function (ECDF), respectively. The coverage of the RF metric is very close to 95 (see Table 2, with n=100), and the rank distribution is approximately uniform on (1, L+1); together, these panels indicate this model is correctly implemented.

Here, there are a few points worth noting. First, for large numbers of species, random tree $\phi_{0}$ is unlikely to share internal nodes with ϕ’s posterior samples (Steel 1988). One way of looking at this involves noting that, if ϕ and $\phi_{0}$ have very many (s) tips, they will share a number of clades that are approximately Poisson distributed with rate $c_{\phi }∕2s$ ($c_{\phi }$ is the number of cherries in ϕ; Bryant and Steel 2009). Given that the expected number of cherries in a random Yule-tree asymptotes to s∕3 for large k’s (McKenzie and Steel 2000), two random Yule trees will asymptote at a shared number of clades that is Poisson distributed with rate 1∕6. One can then see that in the case of Yule trees, ϕ and $\phi_{0}$ have approximately a 85% chance of not sharing any clades. For the reasons above, the RF distance metric may not be so useful.

In the supplement we consider other phylogenetic tree metrics that could be used as an alternative or in addition to the RF distance (Supplementary Fig. 4). Each of these metrics capture different features of parameter space, are computed at varying computational costs (e.g., the ${\text{BHV}}_{0}$ metric is more costly than the RF distance; Supplementary Table 4) and may be more or less useful in revealing problems with a tree model implementation. We leave a more detailed comparison of such tree statistics for a future investigation.

The second validation strategy we introduce allows one to evaluate the coverage of a phylogenetic tree’s topology by looking at the statistics of its clades. The procedure verifies that, across all n independently simulated trees, true clades (i.e., clades present in the simulated trees) are sampled in proportion to their fraction among all clades sampled as frequently. Put differently, one expects that true clades comprise 10% of all clades with posterior support of 0.1, 20% of all clades with posterior support of 0.2, and so on (Supplementary Fig. 7). Importantly, this validation method requires that the tree model only generates trees of the same size, which limits its wide application to all existing tree models. In the supplement we suggest statistical tests for verifying the adherence of a tree model implementation to the aforementioned expectations; we also further discuss the interpretation of this validation method’s output.

### Software

We implemented a suite of methods for automating many of the steps involved in coverage validation and RUV. These methods were developed in Java and integrated into the BEAST 2 platform (Bouckaert et al. 2019). The supplement includes a worked example in which we use the tools available in BEAST 2 and the Experimenter package. Code and a longer tutorial are also available on https://github.com/rbouckaert/DeveloperManual.

## Bayesian Model Validation Guidelines for Developers and Reviewers

In the previous sections, we described and executed two procedures for validating Bayesian models, namely, coverage validation and RUV. Following these procedures should validate any Bayesian model ℳ that (i) can be defined in explicit terms as introduced under the “Probabilistic models” section, (ii) can generate synthetic data, (iii) can be used in statistical inference while having the same exact mathematical form as the model used in (ii) (i.e., can be correctly specified). If a model meets the three aforementioned requirements, it can be shown to be correct by the protocols illustrated in Figure 3, regardless of the nature of its parameter space and its component sampling distributions.

Because the protocols described above provide clear, objective rules for assessing model correctness, carrying out an analysis of coverage and/or of the distribution of parameter value ranks (with respect to their posterior samples) should, on one hand, be a requirement, and on another should suffice for introducing a new Bayesian model implementation.

### Practical Guidelines

Much like in Bayesian statistical analyses, where researchers (ourselves included) employ “default” or “common” priors all too often—as opposed to carefully crafting models to match the uniqueness of their studied system—there is also a danger of being over-prescriptive when recommending validation protocol guidelines. We thus provide below what we deem to be just a rough starting point for those interested in validating or assessing the validation of Bayesian model implementations.

In the examples presented above, we used 100 different data sets (i.e., n=100; Fig. 3), each coming from a unique combination of parameter values drawn from their prior distributions. There is nothing magical about the number 100, however: it is arbitrary, and authors (e.g., Gavryushkina et al. 2014; Gaboriau et al. 2020; Ogilvie et al. 2022) have used it because it is pleasantly round as a denominator, allowing for immediate mental calculation of coverage values. In our experience, n=100 has proven to be a large enough number of simulations for detecting model implementation issues, but lower or greater n’s can be used (the latter being preferred; Table 2), with higher error margins and greater running times as the costs associated to each end of n, respectively.

Two other numbers to consider when carrying out RUV, in particular, are the number of samples to extract from the output of an MCMC procedure (L in Fig. 3), as well as the number of bins when displaying ranks as histograms (middle panels in Fig. 5). First, we recommend the effective sample size (ESS; a quantity well known in Bayesian statistics) of the MCMC output as a ceiling for L—the logic here being that fetching “ESS” equidistant samples from the set of posterior samples will minimize sample autocorrelation. ESS’s of at least 200 have become somewhat of a minimal requirement for Bayesian evolutionary analyses, but larger numbers are preferred.

Given a correctly implemented model and that a 95% confidence interval is used, the heights of approximately 5% of the histogram bars should fall outside the interval. This makes any integer that is greater than (as well as a product of) 20 a convenient number of histogram bins. More generally, bin widths should be adjusted to L so that all bins have at least one, but ideally more rank values falling within its bounds. Empty (or nearly so) bins suggest bins are too narrow or L is too low.

The last and most idiosyncratic feature of a validation experiment to be considered is the ideal size of the simulated data set. Every model is characterized by a data set size beyond which parameters can be estimated with confidence. If much larger, a data set can make statistical inference too slow, if much smaller, certain attractive features of model validation cannot be leveraged, and implementation bugs may remain undetected.

In the case of Bayesian models, too few data points will cause parameter mean posterior estimates to fall along their prior means (see, e.g., λ in Fig. 4), indicating that true parameter values cannot be learned. While the theory behind and interpretation of coverage validation and RUV remain unchanged—that is, (1-α) parameter coverage and uniformity of ranks are still to be expected—one loses the ability to measure inferential accuracy. More troubling, however, is the possibility that implementation bugs hide behind mean posterior estimates indistinguishable from the parameter’s prior mean. We, therefore, recommend data sets be as big as necessary for at least one, but ideally, more parameters (e.g., r and root age in Figs. 4 and 7) to have their values estimated as different from their prior means.

### My Model Implementation Failed Correctness Tests, What Now?

Method developers should expect their software to often fail validation, especially at early development stages, causing the loops in Figure 3 to be visited many times. The validation procedure is almost always arduous and repetitive, but very effective in revealing issues and giving modelers peace of mind when releasing their software. A correctly implemented inference machinery can nonetheless still fail a validation test if there is some unforeseen form of model misspecification (e.g., truncation, see “Scenarios 1” and “Scenarios 3” in Fig. 4). In such cases, a potentially delicate stage of method development begins, when decisions must be made between further testing or software release.

If validation success is marginal or contingent upon a substantially constrained parameter space, or if a Bayesian method has good coverage but fails the demanding RUV (as shown here and elsewhere; McHugh et al. 2022), further simulation experiments might illuminate the nature of the model misspecification and suggest ways to modify the model. For example, developers may want to tweak an aspect of simulation, and then repeat RUV in search for regularities in parameter over- or underestimation (e.g., Section 3 in the supplement). When releasing a method despite validation failure, researchers should in the very least be expected to report all attempts made to validate an implementation, why they seemed to fail, and what biases were uncovered, if any. Ideally, guidelines should be provided for interpreting results obtained with a tool known to be biased.

When confronted with utter validation failure, we urge method developers to resist the temptation of downplaying the importance of the validation effort and instead ask the hard question of whether their models are reasonable in the first place. On one hand, researchers may fail to validate a new model with obvious shortcomings (e.g., Ree and Smith 2008; see also Ree and Sanmartín 2009; Goldberg et al. 2011; Matzke 2022)—or perhaps it is not even clear how to validate the model (e.g., phylogenetic models employing node-age priors; Ho and Phillips 2009)—yet that new model may still improve on the total absence of statistical methods, and ultimately teach us something novel about the natural world.

On the other hand, if large numbers of simulations must be rejected so as to obtain realistic data (or data whose probabilities can be calculated), this could be a sign that the model needs to be modified. Independent implementations that do not pass validation tests provide further evidence that the issue is potentially in the model assumptions themselves. Although historically model design has often gone in the direction of incrementally generalizing existing models (e.g., nested molecular substitution models; Felsenstein 2004), re-imagining the model entirely can sometimes be the best solution.

### Model Characterization

In addition to the model validation we detailed above, there is an infinite number of ways in which a new or published model can have its behavior inspected. Researchers may want to know, given a model, how sensitive parameter estimates are to data set size, prior choice, model complexity, and violation of model assumptions, to name a few. Studies have examined how these factors affect estimation accuracy and precision (e.g., Luo et al. 2023; Zhang et al. 2024), as well as the mixing and convergence of MCMC chains (e.g., Nylander et al. 2004; Zhang et al. 2024). We collectively refer to these examinations as “model characterization”: any analysis of model behavior beyond assessing its correctness. Model characterization is rarely carried out to satisfy the curiosity of the theoretician (but see, e.g., Tuffley and Steel 1997; Steel and Penny 2000); it is instead normally motivated by a model’s empirical applications. These investigations are thus critical for the longevity and popularity of a model, as domain experts will only adopt a model widely if they know when to trust the results and how to interpret them.

It is possible to characterize certain aspects of model behavior while simultaneously verifying its correctness, as discussed in the coverage validation section above. For example, one can observe how accurate parameter estimates are (e.g., if the points in Fig. 4 fall on the identity line) under both correctly and incorrectly specified models. However, the requirement of simulating parameter values from a prior distribution $f_{\Theta }(\cdot )$ during the validation of a model can complicate its characterization. Depending on the characterization experiment’s goals and design, researchers may find themselves rejecting a large fraction of simulated data sets—perhaps because data sets do not resemble those in real life or because they are too large to analyze. But, as we showed, rejecting draws in simulation may then be picked up by the validation protocol as an incorrectly implemented model. This problem can only worsen the more dimensions of parameter space are allowed to vary. In most cases, it may thus make more sense to first verify model correctness by following the procedures we described above, and then characterize model behavior further in a subsequent batch of analyses.

We conclude this section by proposing that scientists contributing or reviewing a new model ask the following question: Is the contribution at hand carrying out an empirical analysis that will specifically profit from scrutinizing model behavior? If not, then model characterization efforts will likely serve their purpose better elsewhere, and profit from being shouldered by the scientific community at large.

## Concluding Remarks

In order to keep up with the large amounts of data of different kinds accumulating in public databases, researchers in the life sciences must constantly update their computational toolboxes. New models are implemented in computational methods every day, but if they are not properly validated, downstream conclusions from using those methods may be void of any significance.

In the present study, we described and executed two distinct validation protocols that verify a Bayesian model has been correctly implemented. Although we looked at examples from evolutionary biology, specifically statistical phylogenetics, these two simulation-based protocols work for any and all Bayesian models.

We further elaborate on the difference between experiments in model validation versus model characterization. Newly implemented models can only profit from validation experiments, which are strictly concerned with theoretical expectations (e.g., about coverage) a model must meet if correctly implemented. Model characterization, on the other hand, is about inspecting model behavior as a variety of data set and model attributes interact; here, exact quantitative predictions may not be theoretically guaranteed. Such experiments are best designed and justified when empirically motivated.

We hope the guidelines described here can enhance both the release rate and standards of statistical software for biology, by assisting its users, developers, and referees in quickly finding common ground when evaluating new modeling work.

## Supplementary material

Data available from the Dryad Digital Repository: https://doi.org/10.5061/dryad.xksn02vpk.
